# Enhancement of Collective Immunity in Tokyo Metropolitan Area by Selective Vaccination against an Emerging Influenza Pandemic

**DOI:** 10.1371/journal.pone.0072866

**Published:** 2013-09-18

**Authors:** Masaya M. Saito, Seiya Imoto, Rui Yamaguchi, Masaharu Tsubokura, Masahiro Kami, Haruka Nakada, Hiroki Sato, Satoru Miyano, Tomoyuki Higuchi

**Affiliations:** 1 Institute of Statistical Mathematics, Tachikawa, Tokyo, Japan; 2 Human Genome Center, Institute of Medical Science, University of Tokyo, Minato-ku, Tokyo, Japan; 3 Division of Social Communication Systems for Advanced Clinical Research, Institute of Medical Science, University of Tokyo, Minato-ku, Tokyo, Japan; 4 Department of Preventive Medicine and Public Health, National Defense Medical College, Tokorozawa, Saitama, Japan; University of Oxford, Viet Nam

## Abstract

Vaccination is a preventive measure against influenza that does not require placing restrictions on social activities. However, since the stockpile of vaccine that can be prepared before the arrival of an emerging pandemic strain is generally quite limited, one has to select priority target groups to which the first stockpile is distributed. In this paper, we study a simulation-based priority target selection method with the goal of enhancing the collective immunity of the whole population. To model the region in which the disease spreads, we consider an urban area composed of suburbs and central areas connected by a single commuter train line. Human activity is modelled following an agent-based approach. The degree to which collective immunity is enhanced is judged by the attack rate in unvaccinated people. The simulation results show that if students and office workers are given exclusive priority in the first three months, the attack rate can be reduced from 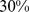 in the baseline case down to 1–2%. In contrast, random vaccination only slightly reduces the attack rate. It should be noted that giving preference to active social groups does not mean sacrificing those at high risk, which corresponds to the elderly in our simulation model. Compared with the random administration of vaccine to all social groups, this design successfully reduces the attack rate across all age groups.

## Introduction

New influenza strains continuously appear via mutations and/or reassortment. Once a new strain acquires stable human-human transmissibility, a pandemic can easily arise since few people will have immunity against that strain. Recently, we experienced the 2009 pandemic. Fortunately, the 2009 flu was low-pathogenic strain, and its social impact was similar to that of seasonal influenza [1], although the symptoms were more severe in young children [2]. However, there is a highly pathogenic strain of avian flu A (subtype H5N1), and sporadic cases of human infection with this strain began to be reported in the late 1990s [3]. If this strain were to acquire stable human-human transmissibility, the resulting pandemic would have dire consequences.

Pioneering studies of the application of agent-based simulation to influenza prevention were performed for Southeast Asia [4], [5] and the US and UK [6]. This research studied the feasibility of containment, that is, preventing the spread from one small population to another within a targeted region/country, as a function of the global reproduction number. We here mention similarity and difference between our model and the ones that were used in these studies. In Ferguson et al., a household for each individual, a single group to which each individual belonged, and the community to which the individual belonged were included in the calculation of the force of infection (FOI). The contribution from the community was modelled by a gravity model, and the corresponding FOI term decreased with the distance from infectious individuals. Instead of the effect of the community, Longini et al. allowed individuals to belong to multiple groups. In our simulation model, the concept of belonging to a group was implemented by the individuals' daily schedule, and the disease was transmitted inside these temporary groups. This approach requires the time step of the simulation to be small (1 minute in our simulation compared to 6 hours in Ferguson et al. and 24 hours in Longini et al.). We took this computationally expensive approach in order to incorporate the effect of individuals moving around the target city via trains.

The present study uses large-scale multi-agent simulations to evaluate the effectiveness of vaccinations in suppressing an influenza epidemic in an urban area. During epidemics inside a city, trains (or other public transport systems) play an important role in the increase in the number of infected people. Trains not only transport people to their workplaces or schools, but also gather them in high-density groups. One impractical approach for suppressing the spread of epidemics is to suspend train services and/or implement furloughs to prevent the concentration of people in places where transmission would be high. However, since these measures are obviously unacceptable due to decreased quality of life and economic impacts, other measures are desired that can be applied without halting the activity of the city. The administration of vaccines to enhance the collective immunity is one means of suppressing the spread of influenza. Since we cannot assume a long period of time from the discovery of the strain to its arrival in any particular region (e.g., the 2009 pandemic strain arrived in Japan two weeks after discovery) and since border quarantine inspections cannot fully detect infected people [7], the administration of vaccine to the residents of the region will be simultaneous with the spread of the influenza. We were interested in determining if the degree of collective immunity could be enhanced if vaccines were distributed preferentially to some selected groups rather than equally to all individuals. To investigate this, we carried out simulations in which different groups received priority for vaccination.

## Method

### Modelling of human activity

We employed a multi-agent simulation approach to describe groups of people who are able to contact each other. Individuals move around different kinds of places in an urban area according to a role-dependent schedule. The disease (influenza) is stochastically transmitted in these local groups, which are formed as a consequence of the actions of individuals.

Our simulator contains several towns in an urban area that are connected by a commuter train line, along which influenza can spread. Each town consists of several types of places and residents. The places include workplaces, schools, homes, and public places, such as shops and parks. Residents are classified into employees, students, and domiciliaries, where domiciliaries referes to individuals who spend the majority of their day in their home (e.g. stay-at-home caretakers, some elderly persons). The members of the first two classes have a specific place where they visit every day and relatively many opportunities to contact with other people. The members of the third class (domiciliaries) spend most of the day in their households and have fewer contacts. The types of places and residents are related by the schedules followed by individuals. For example, if an individual has the role of “employee,” then their schedule requires that they go toward a particular “workplace” at a randomly selected time each morning (for the other elements in schedules, see Section S1.4 of File S1). As a consequence of the evaluation of these schedules for the residents, temporary groups are formed, such as “workplace,” “schools,” and so on. It is assumed at any moment that the disease is transmitted only inside such groups.

**Figure 1:**
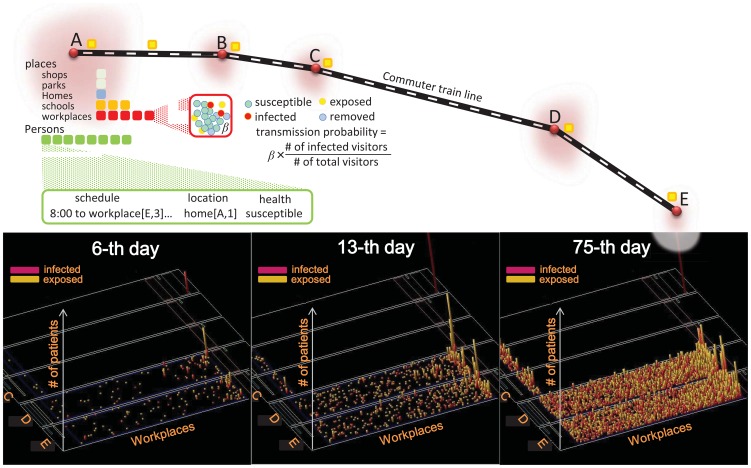
Schematic illustration of the simulator and the model city. Individual activities are explicitly described and infectious transmissions are stochastically evaluated at local places according to the number density of infectious visitors. The bottom three panels show how influenza spreads over many workplaces in a typical baseline simulation run, where the other places are blank due to a small number of patients there.

In this study, the model was constructed to render a simplified Tokyo metropolitan area. The urban area has five towns connected by a commuter train line. A schematic illustration of the model city and the simulation is shown in Fig. 1. For the numbers of residents and schools in towns A–E (Table 1), we used data for the year 2005 as published by the Tokyo metropolitan government [8]. However, the numbers of workplaces are arbitrarily given so as to be concentrated at the central areas (towns D and E) and their scale (the number of employees) is assumed to follow a Pareto law (Fig. S3 in File S1). The bottom three panels of Fig. 1 show how epidemic spread over workplaces. It takes a fortnight or longer for most of workplaces to encounter infectious persons (middle panel) and an epidemic peaks at around the 75-th day (right panel; here a Pareto low distribution of workplaces can be seen). We shall do two different scales of simulations. In full-scale simulations, the numbers of places and residents are the same as Table 1 (the number of agents is 1,246,732; see Fig. S2 in File S1 for age-specific population of each role). In the scaled-down simulations, these numbers are reduced by a factor of one-tenth, namely, the number of agents is 124,673.

**Table 1:** Places and Population in the model city.

town	school	workplace	park	population	shop
A	70	100	2	571,641	100
B	20	100	2	176,866	100
C	12	100	2	138,684	100
D	29	2000	2	314,861	100
E	8	2000	2	44,680	100

### Modelling of Transmission and Vaccine

In our simulation, the health state and the progression of the disease are described in a manner similar to the traditional Susceptible-Exposed-Infectious-Recovered (SEIR) model [9] except that here the disease is stochastically transmitted in local groups. The health status of any individual is described by one of four states: *s* (susceptible), *e* (exposed; infected but not yet infectious), *i* (infected; infected and infectious), and *r* (removed; either recovered or dead).

For the stochastic transition of an individual in state 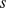 to state 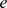, the probability per unit time of the transition is proportional to the density of individuals in state 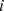 in the same place and the transmission efficiency assigned to that place. The transmission efficiencies of the various types of places are shown in terms of the basic reproduction number in Table 2. Further transitions of each individual's health status to state 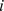 and state 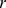 are made with constant probabilities. The inverses of these probabilities are the mean latent and infectious periods and are assigned typical values [10] of 3.5 days and 3 days, respectively.

**Table 2:** Transmission efficiencies of each place type in terms of basic reproduction number.

place	train	school	workplace	household	park	shop	AR
case A	1.5	2.0	2.0	2.0	1.0	0.6	0.30
case B	1.0	2.5	2.5	2.0	1.0	0.6	0.29
case C	1.0	2.0	2.0	4.0	1.0	0.6	0.28

The effect of the vaccine is controlled by two parameters: 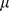, which indicates whether a vaccine enhances the immunity of a dosed person, and 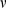, the degree to which their immunity is enhanced. If a vaccine is given, the immunity of the target person is enhanced with probability 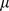, and the degree of immunity is enhanced and the symptoms relaxed by a factor of 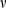. Suppose that 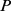 is the probability that an unvaccinated susceptible person transitions into an exposed state. If the person is vaccinated and the vaccine is effective, then the probability is reduced to 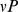 and the average latent period 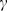 is shortened to 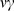. The parameter 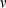 takes a value between 0 (perfect protection) to 1 (useless vaccine). For further details of the implementation, see Section S1.2 of File S1 (Fig. S1 in File S1).

In general, it is not known in which place, households or workplaces/schools, there is a higher likelihood of transmission. We thus considered three representative cases while keeping the resultant global attack rate (AR) around 30%: (A) household dominant, (B) workplace/school dominant, and (C) all three places being equal. In order to inform relative importance, we show the number of transmission in each kind of places in Table 3. Previous studies [4], [5], [11], [12] devoted to agent-based influenza simulations assigned 33% to the AR; this was derived from a retrospective analysis of the 1957–1958 pandemic. It would be useful to compare the value we used for the transmissibility with the values used in previous works. Because we used a different transmission model, however, we need to first examine the corresponding model parameters. Let us consider a situation in which a person remains stationary and receives 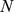 visitors, 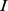 of whom are infectious. In these studies, the transmission process is governed by two parameters, the transmission probability 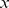 per contact and the number 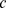 of persons with whom a person has contact on a given day. The probability 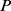 with which the person transfers to the exposed state is given by
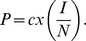
In our model, the counterpart of 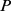 is given by
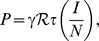
where 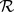 is the site-specific transmission efficiency in terms of the reproduction number and 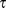 is the time spent visiting that location. Comparing the two forms for 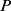, we obtain the formula 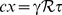 to relate the parameters of previous studies with ours. Table 4 compares the transmission efficiencies of case A in Table 2 with those of Cooley et al. [12], which studied the importance of transmission in subways in New York City. Our values for transmission in workplaces and households are comparable to the estimations of Cooley et al.; their values for commuter and noncommuter passengers differ greatly, and it is difficult to say anything beyond that our value is between these two.

**Table 3:** Number of transmissions in each place type in baseline simulations.

place	train	school	workplace	household	park	shop
case A	13498	2558	12623	10537	85	413
case B	8736	2816	15330	10052	51	395
case C	7777	2145	10597	12663	93	363

**Table 4:** Comparison of our transmission efficiencies with those of Cooley et al. [12].

place	train 1	train 2	workplace	household
*P* (this paper)	0.1667		0.3333	0.3333
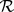	3		2	2
*τ*	2 hr		12 hr	12 hr
*P* (Cooley et al.)	1.9481	0.0324	0.2116	0.3688
*x*	0.0575	0.0048	0.0575	0.4
*c*	33.88	6.75	3.68	0.922

Cooley et al. assigned different values of 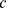 and 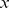 to commuter passengers (train 1) and noncommuter ones (train 2). The value of 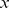 assigned to adult-adult contact in households is used here, while different values were assigned to adult-children and child-child contacts in the original work.

In a crowded train, one passenger may approach very close to another, and there is then a higher probability than usual that the disease will be transmitted. In order to incorporate this effect, we allowed the transmission efficiency on trains to increase with the mean person-person distance, which is calculated from the number of passengers. Based on studies of epidemics [13] and aerosol dynamics [14],[15], droplet transmission is very effective at distances of up to 1 meter, but may still be possible up to a distance of 3 meters [16]. Based on this, we employed a transmission efficiency function that has a cutoff at 3 meters. The reproduction number in trains thus takes the value of 1.0 at this cutoff and increases up to 1.93, as shown in Table 2 (see File S1, Section S1.3 for further details).

### Baseline epidemic simulation

**Figure 2:**
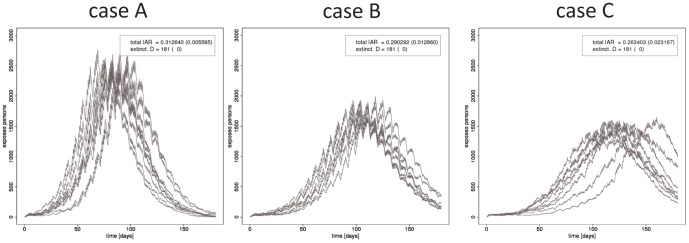
Baseline (without vaccination) simulation run. Variation of the numbers of residents in states 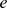 is shown for cases A, B and C of transmissiblity.

As a baseline for the later evaluation of vaccination programs, we calculated the pattern in which influenza spread in the model urban area when there was no intervention (See Table S1 in File S1 for a complete list of parameters to configure simulation). This area was an abstraction of the towns along a commuter rail in the Tokyo metropolitan area. We assumed the initial number of infected people was 600 to ensure an outbreak would arise, that is, we did not consider cases in which the introduced strains became extinct. Figure 2 shows the spread pattern for a typical simulation run of case A. The epidemic peak occurs on approximately the 75-th day (left panel). We modelled that office workers and students remained at home on nights and weekends. This effect was reflected in the small daily but large weekly oscillations in the number of patients. The proportion of residents who are eventually infected is called the illness *attack rate* (AR). The cumulative number of infected people, shown in the middle panel, indicates that the AR is 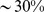 in the baseline simulation.

### Calibration of vaccine strength

Prepared vaccines vary greatly in effectiveness depending on the target virus. Here, we calibrate the effect of the vaccine in simulations to reproduce an efficacy value estimated by retrospective studies for real seasonal influenza [17], [18], [19]. The efficacy is defined in terms of the ARs of the vaccinated and unvaccinated groups [20],
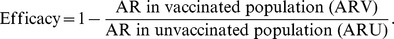
In real investigations, the ARV and ARU are calculated for different populations that satisfy the definitions, whereas in the simulations these can be calculated from two simulation runs, with vaccination and without vaccination, respectively, for a chosen small population that does not affect the collective immunity of the entire city (we set this to be 0.4% of the city residents).

Table 5 shows the efficacy calculated in this manner for different settings of vaccination strength. In this table, 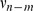 denotes the value of 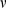 for people aged 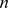 to 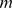 years. In this experiment, the age-based values 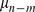 of the probability 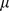 were chosen to be 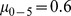, 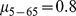, and 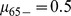. Based on studies [17], [18], [19] that state that the efficacy is approximately 60% in adults, we set 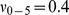, 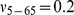, and 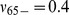.

**Table 5:** Values of vaccination strength and resultant efficacies.

*ν*_1–5_	*ν*_5–65_	*ν*_65–_	efficacy	illUnc	illVac
0.2	0.1	0.2	0.7264	0.5338	0.1460
0.4	0.2	0.4	0.6073	0.5338	0.2096
0.8	0.4	0.8	0.4533	0.5338	0.2918
0.9	0.5	0.9	0.3844	0.5338	0.3286

Attack rates in the unvaccinated and the vaccinated populations are denoted by illUnc and illVac, respectively.

## Results

We studied the performance of vaccination programs for the enhancement of collective immunity. Each program was defined by the target group and the coverage of the population of the urban area. The performance of each program was evaluated by the reduction in the AR of the unvaccinated population. For target groups, we considered arbitrarily chosen residents (Arb), employees (Emp), students (Stu), the combined group of students and employees (StuEmp), domiciliaries (Dom), train passengers (Tr), and the subset of StuEmp who use trains to get to their offices or schools (TrStuEmp). For each target, we carried out simulation runs in which 16%, 32%, and 48% of the chosen group were vaccinated. We assumed that vaccines could be distributed to 48% of the residents in three months; we also considered half and twice this reference rate. In all three cases, we assumed for simplicity that the vaccine stockpile was uniformly distributed. This reference speed was chosen according to the 2009 vaccination schedule in Japan [21]. In this schedule, domestic production covers 45% of the Japanese population in six months, from October to March. The priority groups included pregnant women, individuals with diseases, children, and elderly persons. The rest of the population was covered by the remainder of the domestic vaccine, and supplemented by imported vaccine. We note here the discrepancy between the plan mentioned above and the estimation [22] of the actual number of vaccines. Looking at Table 6, we see that the planned rate and the actual rate are similar until the middle of December, after which the actual rate is less than the planned rate. According to reports of a learned discussion [23], 2009 flu may influence people hesitate to be vaccinated and so there was an oversupply after January.

**Table 6:** Schedule of domestic vaccine supply for Japan in the 2009/2010 season and estimation of actual consumed courses.

Month	2009–10		11		12			2010–01		2		3		Total
Period	I	II	I	II	I	II	III	I	II	I	II	I	II	
Plan(×10^4^)	-	118	134	364	452	572	515	459	659	649	557	349	560	5,388
Actual(×10^4^)	-	158	303	142	418	627	227	162	170	-	-	-	-	2,207

**Figure 3:**
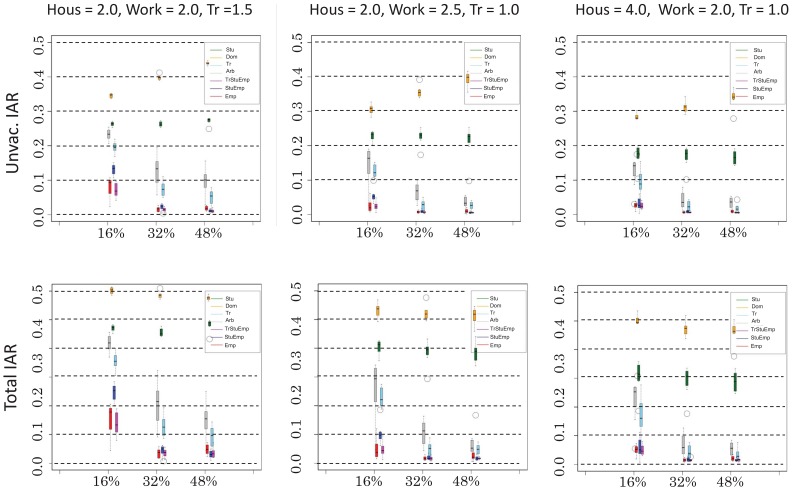
Dependence of the illness attack rate (AR) on the target selection. The abscissa denotes the proportion of vaccinated people and the ordinate denotes the AR in the unvaccinated population (left panel) or of all residents (right panel). For each target, the coverage is chosen to be 16%, 32%, or 48% in terms of whole the residents, and the speed of administration of vaccine is chosen so that 16% of the residents are distributed in 30 days.

Figure 3 shows the AR in the unvaccinated population as a function of the number of vaccinated people. In the top panels, the denominator of the AR is the unvaccinated population (hereinafter this rate is called the unvaccinated AR), and the denominator in the bottom panels is the population of the entire city (hereinafter this rate is called the total AR). For each different vaccination program, we carried out eight runs, each with a different random initial distribution of infected individuals. Administration of the vaccine to employees was the most effective way to reduce the AR, followed by vaccinating students and domiciliaries. This is reasonable since office works are the most active residents. Targeting office workers was advantageous for all the different cases of transmission efficiencies, including case C, which assumed that households have higher transmission rates than do workplaces. If office workers, corresponding to 32% of the entire population, are vaccinated, the total AR is reduced to 2%. Observing the number of infected individuals over time, we find that transmissions are suppressed and become sporadic. When other groups are targeted instead, there is some reduction in transmission but not to this extent.

Vaccination of domiciliaries produced a very limited effect. The total AR was almost the same as that of the baseline. Note that the unvaccinated AR was larger than the baseline AR, and it even increased with increased vaccine coverage due to changes in the denominator. As the number of vaccinated domiciliaries increases, the proportion of office workers in the unvaccinated group increases. This increases the unvaccinated AR.

**Figure 4:**
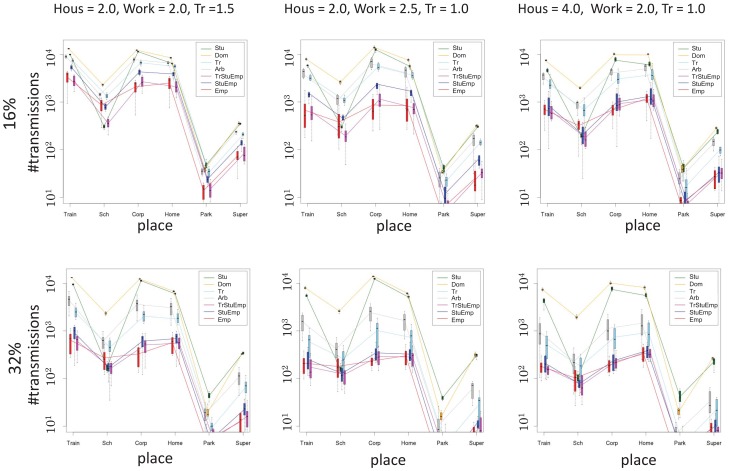
Number of transmissions in each place type in simulations with vaccination.

Targeting students had a slightly better effect, but it did not bring benefits to unvaccinated individuals. When students making up 16% of the city residents were vaccinated, the total AR was reduced to 20–25%, depending on the efficiency of transmission. The transmission rate in the different types of location provides information about the extent of benefit to unvaccinated individuals, as shown in Fig. 4. Vaccinating students somewhat reduced transmissions in schools, comparable to the effect in offices when office workers were targeted. The number of transmissions in other locations, however, remained near the baseline level.

Vaccinating arbitrarily chosen people provided an intermediate effect. Unlike with students, the AR can be further reduced by extending the coverage. Targeting random train passengers for vaccination was slightly better than a fully random program. This is because the proportion of office workers is larger in the train passenger group than in city as a whole.

Prioritizing active members, as suggested by the simulation results, is contrary to conventional approaches, which prioritize high-risk individuals (e.g., the elderly and people with disease) followed by medical practitioners. One may wonder if prioritizing office workers would disadvantage high-risk individuals, but as we show in Table 7, prioritizing active members results in a smaller attack rate of the elderly than does random vaccination.

**Table 7:** Age-based AR for each vaccination target in case A.

Target	Age 0–5	Age 5–65	Age 65–
coverage 16%			
Arbitrary	0.205 (0.014)	0.240 (0.018)	0.076 (0.006)
Domiciliary	0.301 (0.007)	0.347 (0.006)	0.106 (0.005)
Student	0.142 (0.008)	0.276 (0.007)	0.083 (0.004)
Employee	0.100 (0.037)	0.086 (0.031)	0.029 (0.011)
coverage 32%			
Arbitrary	0.103 (0.037)	0.118 (0.043)	0.036 (0.015)
Domiciliary	0.295 (0.014)	0.340 (0.006)	0.094 (0.003)
Student	0.124 (0.007)	0.268 (0.008)	0.080 (0.003)
Employee	0.029 (0.015)	0.020 (0.009)	0.007 (0.003)
coverage 48%			
Arbitrary	0.079 (0.032)	0.087 (0.035)	0.025 (0.010)
Domiciliary	0.290 (0.008)	0.335 (0.004)	0.089 (0.003)
Student	0.146 (0.011)	0.282 (0.012)	0.085 (0.006)
Employee	0.038 (0.013)	0.028 (0.010)	0.009 (0.003)

^*^All data are given as average and standard deviation (in paren.) of 64 simulation runs.

**Figure 5:**
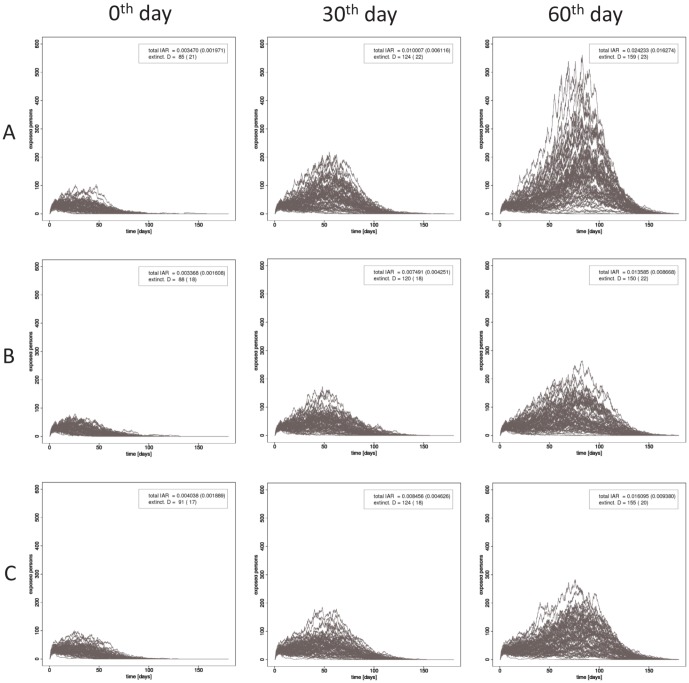
The dependence of 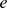 on the onset of vaccination. To show the effect of the selection of initial patients and the stochasticity of transmission, 64 runs (8 different choices of initial patients and 8 different random seeds) are carried out in each case. The administration covers all residents in 180 days and vaccinations are carried out in the order of TrStuEmp, the rest of StuEmp, and finally Dom.

We have shown the results of vaccinating a single group. We demonstrated that, with the same distribution speed (96% of residents are covered in 180 days), targeting offices workers for vaccination resulted in the greatest reduction in the AR, followed by targeting students and then domiciliaries. Through this same demonstration, we also evaluated the degree to which the collective immunity would be degraded due to a delay in the design or manufacture of a vaccine. Figure 5 shows the number of patients over time for three different transmission efficiencies and for the 0-th, 30-th, and 60-th day as the start date of the administration of vaccine. Each panel corresponds to 64 simulation runs (8 different choices of initial patients and 8 different random seeds). The differences among the three cases is small. A small epidemic occurred (AR is 0.3–0.4%) and about 90 days were needed for extinction of the transmission chains, even if the administration of vaccine was begun at the start of the simulation. If the initiation of vaccination was delayed, the scale of the outbreak increased: AR was 1–2% and the extinction was delayed until the 150-th day. This reduction of the AR to a small value despite a delay indicates that vaccination is sufficient to protect against low-pathogenic strains, whereas aggressive intervention (e.g., school closure and traffic blockades) should be combined with vaccination against highly pathogenic strains, since vaccination alone will not lead to early extinction.

## Discussion

We have showed that the AR can be reduced to 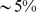 or less if students and employees are intensively vaccinated in the first 90 days. This is the result of an intervention program that relies solely on vaccinations, and the AR can be further reduced by individual protection efforts (e.g., wearing masks and avoiding crowded places). If the encountered virus is not highly pathogenic, this value is acceptable. In this case, the goal of intervention is to avoid an excess of patients going to medical practitioners. However, early extinction of transmission chains is required in highly pathogenic cases, and vaccination alone is not sufficient. To achieve early extinction by using only the collective immunity induced by vaccinations, administration of the vaccine would need to be carried out at least three times as fast as the typical speed.

It would be worthwhile to interpret our simulation results along with the situation of the 2009 pandemic (2009pdm) in Japan. Inaida et al. [24] studied the 2009 pandemic geographically and temporally in four metropolitan areas of Japan, analysing time courses obtained from about 5,000 sentinel observation points. The 2009pdm spread similarly in four metropolitan areas and the growth of the number of patients is relatively rapid compared to the surrounding rural areas. However, a correlation between the AR and the rate of large families was recognized in Tokyo and Nagoya, but not in Osaka and Fukuoka. Different transmission models of households for central and suburb areas may need to be introduced into the simulation, since such large families mainly exist in suburban areas.

Our simulations indicate that a two-month delay in vaccination retains an effect on the enhancement of collective immunity to a certain extent. Retracing the timeline of the 2009pdm, we know that this delay is allowable. The analysis of Inadia et al. [24] showed that 2009pdm spread sporadically in Japan during its early stages. The pandemic virus landed during the end of April to the middle of May. The number of reported cases per sentinel site exceeded unity, which is used as a working definition of the onset of an epidemic, in the beginning of August and exhibited further slow growth for more than 6 weeks. The distribution of vaccines began in October. Therefore, the delay of vaccine distribution from the onset of the epidemic was around one month. Nonetheless, the sporadic transmission was perhaps due to the onset in summer, which is unlike seasonal influenza.

The vaccination plan [21] in 2009/2010 season gave a higher priority to certain groups including pregnant women, individuals with underlying diseases, children, and elderly persons. The rest of the population was intended to be covered by the remainder of the domestic vaccine, and supplemented by imported vaccine. The estimated numbers of courses distributed to respective groups [22] in November and December indicate that the distribution of vaccines in the early stage was actually focused on the elderly and high-risk persons as had been planned. In November, the number of distributed courses was 920,000 to people with underlying disease, 1,335,000 to people without underlying diseases and 1,011,000 to the age group above 65, respectively. In December, these numbers were 1,468,000, 3,956,000 and 2,224,000, respectively, where distribution to small children (≤8 years) contributed to a large number of people without underlying disease receiving the vaccine in December. The distribution to the age group of 9–65 is almost the same in these two months (73 and 82.6 thousands, respectively). From this document, we also know that the planned rate and the actual rate are similar until the middle of December, after which the actual rate is less than the planned rate. According to reports of a learned discussion [23], 2009 flu may have influenced people to avoid vaccination, resulting in an oversupply after January.

## Supporting Information

File S1 **Enhancement of collective immunity by selective vaccination against an emerging influenza pandemic.** Contains Figure S1, Figure S2, Figure S3, and Table S1. **Figure S1. Pseudo code of a single step of the simulation.** All information on the simulated urban area is contained in the structure instance city, which is located in shared memory. Iterations and branches are in a Fortran-like code, but the structure name and its field are split by a dot. Simulations are carried out in parallel based on OpenMP, and iterations marked by !OMP DO are adequately split by the compiler and carried out in parallel. Calculation of the transition probability places{key = v}.pr is implemented to conform to the diagram of Eq. (2). **Figure S2. Age-specific distribution of the population of Tokyo in 2005.** We sampled from this distribution to obtain the ages of individuals, and their roles were assigned according to their ages. The proportions of roles in the simulation are represented by different colors (blue: students, red: employees, and yellow: domiciliaries). **Figure S3. Distribution of corporation sizes.** The rank in the corporation size versus the number of employees. **Table S1. List of parameters configuring simulation.**
(PDF)
