# Supplementary material for: Enhancement of Collective Immunity in Tokyo Metropolitan Area by Selective Vaccination against an Emerging Influenza Pandemic
Source: PLoS One. 2013 Sep 18;8(9):e72866. doi: 10.1371/journal.pone.0072866 (PMC3776821; doi:10.1371/journal.pone.0072866)
Supplement: File S1 — Enhancement of collective immunity by selective vaccination against an emerging influenza pandemic. Contains Figure S1, Figure S2, Figure S3, and Table S1. Figure S1. Pseudo code of a single step of the simulation. All information on the simulated urban area is contained in the structure instance city, which is located in shared memory. Iterations and branches are in a Fortran-like code, but the structure name and its field are split by a dot. Simulations are carried out in parallel based on OpenMP, and iterations marked by !OMP DO are adequately split by the compiler and carried out in parallel. Calculation of the transition probability places{key = v}.pr is implemented to conform to the diagram of Eq. (2). Figure S2. Age-specific distribution of the population of Tokyo in 2005. We sampled from this distribution to obtain the ages of individuals, and their roles were assigned according to their ages. The proportions of roles in the simulation are represented by different colors (blue: students, red: employees, and yellow: domiciliaries). Figure S3. Distribution of corporation sizes. The rank in the corporation size versus the number of employees. Table S1. List of parameters configuring simulation. (PDF) [file pone.0072866.s001.pdf]

Enhancement of collective immunity by selective vaccination against an emerging influenza pandemic — Supplementary Information —

July 17, 2013

S1 Description of Simulator

S1.1 General description of data structure and behavior

The simulator developed for the present work is designed to study how an infectious disease spreads in a single urban area. We begin the description of the simulator by viewing the single data structure, which holds all the information for the simulation. We denote the single instance of the structure by city and similarly use bold Roman letters to refer to any items in the computer. The simulated urban area consists of towns and railway lines connecting them, and each town consists of places and residents. The structure of the places has fields for the type of place, the transmissibility parameter β, the numbers of people in various health states who visit, and the transition probabilities between health states. The possible types of places are *corporation*, *school*, *shop*, *park*, *home*, and *train*. Users can characterize the type of places by properly configuring the value of β. A resident has fields for role, health state, place currently visiting, candidates for places to be visited, and a schedule. The health status is defined as conforming to the SEIR macro simulation model[12]. There are four possible states: s means the individual is susceptible to the virus, e means the individual is infected without being contagious, i means the individual is infected and contagious, and r means the individual is either recovered and immune or dead. Individuals can have the role of *employee*, *student*, or *domiciliary*. The simulator is equipped with predefined schedules for each of the roles (the behaviors for each role will be described below). At the scheduled time, the location of each individual is changed instantaneously if the move takes place inside the currently visited town, but if transportation by train is required, the individual remains in place until a train arrives or remains on the train until the journey is completed.

The components mentioned above are combined to form a single step of the simulator shown in Fig. S1. This procedure processes each person in each town. Since determining the movement and the health status for each individual can be performed independently, these operations are carried out in parallel, although the update of the transition probabilities should be atomic (the operations marked with a star in Fig. S1); that is, consecutive operations assigned to a process element are performed, and the rest of the process elements must pause until the operations are completed.

S1.2 Infectious transmission

A single step of the simulation is carried out by updating the currently visited location and the health status of each individual. First, we consider simulations without vaccination. Suppose that the simulator is currently processing an individual who visits a location where the numbers of visitors in total and in state i are N and I, respectively, and the transmissibility parameter is β. Then, the transmission and

```
do area ∈ city.areas
    !$omp do
    do person ∈ area.persons,
        following to person.schedule, change person.visit.
        if person.visit changes from v to v' (v ≠ v') then
            let h = person.visit
            !$omp critical
            decrement     area.places{key=v }.nVisitors{key=h}.
            recalculate   area.places{key=v }.pr.
            Increment     area.places{key=v'}.nVisitors{key=h}.     ★
            recalculate   area.places{key=v'}.pr.
            !$omp end critical
        end if
        change person.health according to probability area.places{key=v}.pr.
        if person.health changes from h to h' then
            let v = person.visit
            !$omp critical
            decrement     area.places{key=v}.nVisitors{key=h }.
            Increment     area.places{key=v}.nVisitors{key=h'}.     ★
            recalculate   area.places{key=v}.pr.
            !$omp end critical
        end if
    end do
    !$omp end do
end do
```

Figure S1: Pseudo code of a single step of the simulation. All information on the simulated urban area is contained in the structure instance city, which is located in shared memory. Iterations and branches are in a Fortran-like code, but the structure name and its field are split by a dot. Simulations are carried out in parallel based on OpenMP, and iterations marked by !OMP DO are adequately split by the compiler and carried out in parallel. Calculation of the transition probability places{key=v}.pr is implemented to conform to the diagram of Eq. (2).

progress of the disease are simulated through a stochastic update of the health status of this individual with probabilities given by the diagram,

$$s \xrightarrow[\beta I/N]{} e \xrightarrow[\alpha]{} i \xrightarrow[\gamma]{} r \ , \tag{1}$$

where the formula accompanying an arrow expresses the transition probability per unit time from the source to the destination. This definition of transition probabilities realizes the variation in the number of people in each state during an infinitesimal time so that the expectation among stochastic variants agrees with the variation of the corresponding variable in the SEIR model.

Simulations with vaccinated people require more states and modifications in the transition probabilities. We thus add state v to the possible health states and give probability μ and hyposensitization parameter $\nu \in [0, 1)$ to the vaccinated people. With the new state v and parameters μ and ν, the transition probabilities are modified as

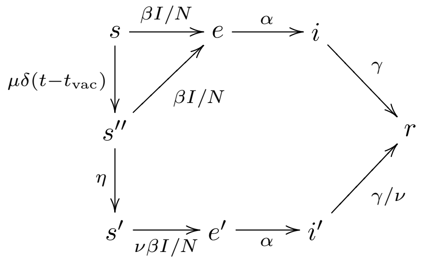

. (2)

The primed states have a meaning similar to that of their unprimed version, but the transition probabilities are modified due to the effect of the vaccine. For the susceptible state, two additional states s'' and s' exist. Both states mean that a vaccine has been administered; the vaccine is not active in s'' but is active in s. The vaccine provided may or may not match the actual virus, depending on both the vaccine design and the individual's immune system. To describe this in the simulator, we decided with probability ν that the dosed vaccine protects the target person. The inverse of the transition probability from s'' to s' is the latent period of activation. The transition from s' to i' is less probable than is that from s to i. This means that the activated vaccine protects a person from being infected by contagious people. The transition from i to r, in turn, is more probable, which reflects that the infectious period is shortened by the vaccine.

S1.3 Enhancement of transmissibility in crowded trains

Trains are different from other places in that the transmissibility may change a great deal due to the number of passengers in the train cars. To incorporate this in our model, we first estimated the mean distance between passengers from the number of passengers in cars, and then calculated the transmissibility as a function of the mean distance.

In typical train cars, the floor area is approximately 60 m^2 ($\approx$ width 2950 mm $\times$ length 200,000 mm), and the standard capacity is approximately 150 people. Based on these parameters, we related the occupancy σ to the mean distance D as

$$D/[\mathrm{m}] = \sqrt{\frac{60}{150\sigma}} \approx \frac{0.63}{\sqrt{\sigma}}. \tag{3}$$

We obtained the maximum number of passengers in one train car by presimulation and noted this number was twice the standard capacity ($\sigma = 2$). The reason we did not calculate the mean distance directly from the number of passengers was to enable the scaling of the city population without changing the frequency

of the train service. Transmissibility significantly increases if D becomes smaller than a critical value D_0. We modelled this by assuming that the reproduction number $\mathcal{R}$ in a train follows a Yukawa-type potential,

$$\mathcal{R}(D) = \mathcal{R}_0 \frac{\exp(-D/D_0)}{D},$$

where $D_0 = 3$m are used and values of baseline $\mathcal{R}_0$ are used in Table 2 of the main text for the description of the reprodution number of trains.

S1.4 Scheduling

The schedules of individual people are decided at midnight every day by the schedule generators prepared for the respective roles.

Employee The mandatory tasks on weekdays are a trip from home to the corporation and a return trip. The scheduler randomly chooses from 6:00–10:00 for the former and from 18:00–22:00 for the latter. Additionally, employees may visit other corporations. If employees have two or more corporations as candidate visiting places, then the generator takes some or all of them and assigns visiting times from 10:00–16:00 and visiting durations from 30 min–2 hours. On days off (Saturday and Sunday), schedules are configured so that employees randomly wander their candidate visiting places.

Student Student schedules are similar to those of employees. However, the trip to school begins from 6:00–8:00 and the return trip is from 16:00–19:00. Some students return to school in the evening, going there from 19:00–20:00 and returning from 21:00–22:00.

Domiciliary This role includes preschool children and retired people. Domiciliaries' visiting places include at least one supermarket located in the town in which they live, and optionally other supermarkets and parks, which may be outside their home town. Domiciliaries are scheduled to visit a supermarket for 1 hour during 10:00–20:00 as a mandatory task, and optionally to visit other candidate places following the same rule.

S2 Configuration of the simulator in the present study

S2.1 Model city

We employed a model urban area for which the number of residents and places are given in Table S1. This model is based on the five towns along an arterial railway line (Chuo Line) going east and west in the city of Tokyo. We used the actual population by 5-year age groups, as provided by the Tokyo metropolitan government for 2005[21]. The distribution is shown in Fig. S2. We sampled from this distribution and then assigned roles according to the following rules: (a) All individuals aged ≤ 22 are students, (b) 70% of the individuals in the range $22 <$ age ≤ 60 are employees, and (c) the remaining 30% of the individuals in the range $22 <$ age ≤ 60 and all individuals with age > 60 are domiciliaries. Homes were constructed by randomly combining several people chosen from the population constructed from these statistics. The number of schools in each town was also taken from these statistics. The number of corporations, however, was arbitrarily chosen so that they were concentrated at the central towns D and E and the scale of the corporations follows a Pareto distribution.

To construct corporations that follow a Pareto distribution, we began by randomly assigning employees to corporations. Suppose that N corporations follow a (truncated) Pareto distribution. Then, the number

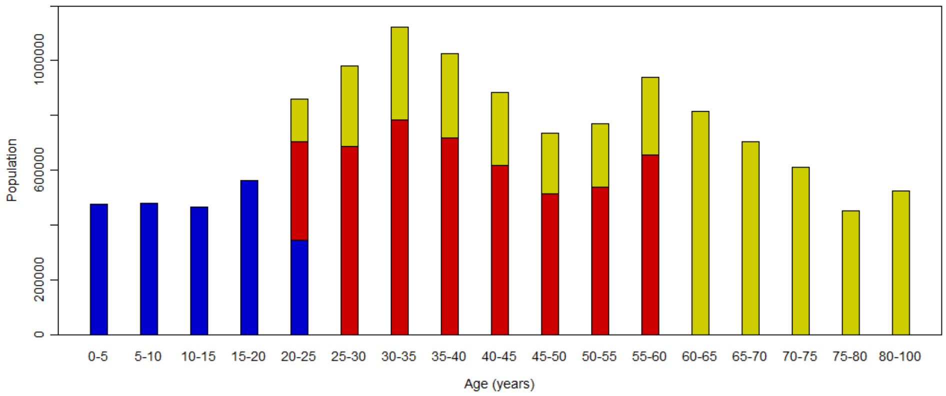

Figure S2: Age-specific distribution of the population of Tokyo in 2005. We sampled from this distribution to obtain the ages of individuals, and their roles were assigned according to their ages. The proportions of roles in the simulation are represented by different colors (blue: students, red: employees, and yellow: domiciliaries).

of corporations, whose scale factor for the number of employees is between x and $x + dx$, is proportional to

$$q(x) = \frac{\beta\alpha^\beta}{x^{\beta+1}} \quad (\alpha \le x \le \gamma),$$

where the lower and the upper limits of the scale factors α and γ are chosen later. The cumulative $Q(x)$ is given by

$$Q(x) = \int_\alpha^x q(x)dx \quad (\alpha \le x \le \gamma).$$

Some discretization is necessary to actually construct a corporation. To do this, we took the scale factor x_y of the y-th ($y = 1, \cdots, N$) corporation from the smallest one, such that

$$\int_{x_{y-1}}^{x_y} q(x)dx = \frac{1}{N}Q(\gamma), \quad \text{or equivalently,} \quad \int_{x_0}^{x_y} q(x)dx = Q(x_y) = \frac{y}{N}Q(\gamma), \tag{4}$$

where $x_0 \equiv \alpha$ and $x_N \equiv \gamma$. Then, we can construct corporations by randomly drawing the index of the corporation, y, with the probability $p(y)$ proportional to x_y, and assigning a person to corporation y, where we have

$$p(y) \propto x_y = Q^{-1}\left(\frac{y}{N}Q(\gamma)\right) \quad (0 < y \le N).$$

The inverse transform sampling enables us to sample y, that is, $y \sim p(y)$ if we set $u \sim U[0,1)$, $P(y) = \int_0^y p(y')dy'$ and $y = P^{-1}(u)$. The actual form for transforming u to y is

$$y = N\left[1 - \left(1 - (\beta - 1)\frac{uP_1}{\alpha\beta}\right)^{\frac{\beta}{\beta-1}}\right] \Bigg/ \left[1 - \left(\frac{\alpha}{\gamma}\right)^\beta\right] \quad \text{with} \quad P_1 = \frac{\alpha\beta}{\beta - 1}\left[1 - \left(\frac{\alpha}{\gamma}\right)^{\beta-1}\right]. \tag{5}$$

The size distribution of the 2,000 corporations obtained in the simulations is shown in Fig. S3. The largest corporation had 2,306 employees and the smallest had 30.

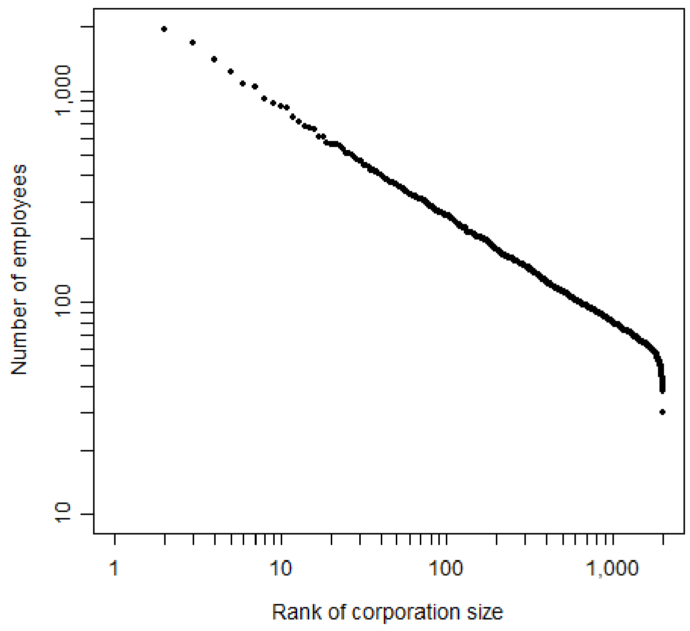

Figure S3: Distribution of corporation sizes: The rank in the corporation size versus the number of employees.

S2.2 Transmissibility and progress of disease

The magnitude and the differences in the transmissibility of disease among the different types of locations are controversial. In the above discussion, we assigned the transmissibility of disease in terms of the reproduction number for location types as follows: corporations (3.0), schools (3.6), hospitals (2.4), homes (2.4), parks (1.0), and shops (0.6).

The parameters α and γ give the inverses of the mean latent and the mean infectious periods, respectively. We set $\alpha^{-1} = 3.5$days and $\gamma^{-1} = 3$days. The entire illness period $\alpha^{-1} + \gamma^{-1} = 6.5$days is reasonable as a representative value for influenza[20]. These values ultimately determine the timescale for the spread of influenza, and the fact that the peak occurs at around the 75-th day in the unvaccinated case also supports this selection.

References

[1] Van Kerkhove, M. D., Vandemaele, K. A. H., Shinde, V., Jaramillo-Gutierrez, G., Koukounari A, et al. Risk Factors for Severe Outcomes following 2010 Influenza A (H1N1) Infection: A Global Pooled Analysis. *PLoS Med* **8**(7) (2011): e1001053. doi:10.1371/journal.pmed.1001053

[2] Sha, L. et al. Analysis of clinical manifestations of hospitalized children infected with seasonal influenza A virus and 2009 novel influenza A (H1N1) virus in Beijing. *Chinese Journal of Pediatrics* **49**(7), 539–544 (2011).

[3] World Health Organization. H5N1 avian influenza: timeline of major events. *Global Alert and Re-*

Table S1: List of parameters configuring simulation

- Mean latent period: $\alpha^{-1} = 3.5$ days
- Mean infectious period: $\gamma^{-1} = 3.0$ days
- Reproduction number $\mathcal{R}$ of each place kind

place	train	school	workplace	household	park	shop	AR
case A	1.5	2.0	2.0	2.0	1.0	0.6	0.30
case B	1.0	2.5	2.5	2.0	1.0	0.6	0.29
case C	1.0	2.0	2.0	4.0	1.0	0.6	0.28

  Note: reproduction number $\gamma\mathcal{R}$ is related to transmission efficiency β, where $\beta = \dot{S}/SI$ with the numbers of susceptible S and infectious I persons.

- Places and Population in the model city

town	school	workplace	park	population	shop
A	70	100	2	571,641	100
B	20	100	2	176,866	100
C	12	100	2	138,684	100
D	29	2000	2	314,861	100
E	8	2000	2	44,680	100

- The number of initial infected people: 600
- Distribution of corporation sizes
  Shown in Fig. S3. Parameters of Pareto distribution are $\alpha = 1,\ \beta = 2,\ \gamma = 100$.

sponse (GAR) 14 July 2011, 1–50 (2011).
http://www.who.int/csr/disease/avian_influenza/ai_timeline/en/index.html

[4] Ferguson, N. M. et al. Strategies for containing an emerging influenza pandemic in Southeast Asia. *Nature* **437**, 209–214 (2005).

[5] Longini, I. M. Jr. et al. Containing Pandemic Influenza at the Source. *Science* **309**, 1084–1087 (2005).

[6] Ferguson, N. M., Cummings, D. A. T., Fraser, C., Cajka, J. C., & Cooley, P. C. Strategies for mitigating an influenza pandemic. *Nature* **442**, 448–452 (2006).

[7] Sato, H., Nakada, H., Yamaguchi, R., Imoto, S., Miyano, S., & Kami, M., When should we intervene to control the 2009 influenza A(H1N1) pandemic? *Euro Surveill.* **15** (1) (2010).
Available from: `http://www.eurosurveillance.org/ViewArticle.aspx?ArticleId=19455`

[8] Feigin, R.D. et al. Epidemic meningococcal disease in an elementary-school classroom. N. Engl. J. Med. **307**, 1255–1257 (1982).

[9] Dick, E.C., Jennings, L.C., Mink, K.A., Wartgow, C.D., Inhorn, S.L., Aerosol transmission of rhinovirus colds. J. Infect. Dis. **156**, 442–448 (1987).

[10] Duguid, J.P. The size and duration of air-carriage of respiratory droplets and droplet nucleii. J. Hyg. (Lond) **44**, 471-479 (1946).

[11] Siegel, J.D., Rhinehart, E., Jackson, M., Chiarello L., the Healthcare Infection Control Practices Advisory Committee, 2007 Guideline for Isolation Precautions: Preventing Transmission of Infectious Agents in Healthcare Settings.

[12] Kermack, W. O., McKendrick, A. G., Contributions to the mathematical theory of epidemics. *Proc. Royal Soc. Ser. A.* **115**, 700–721, (1927).

[13] Saito, M. M. et al. Extension and verification of the SEIR model on the 2009 influenza A(H1N1) pandemic in Japan. *Mathematical Biosciences* (under review).

[14] Jefferson, T., Rivetti, A., Harnden, A., Di Pietrantonj, C., & Demicheli, V., Vaccines for preventing influenza in healthy children (Review). *Cochrane Database of Systematic Reviews*: Reviews 2008 Issue 2 John Wiley & Sons, Ltd Chichester, UK DOI: 10.1002/14651858.CD004879.pub3

[15] Jefferson, T. et al. Vaccines for preventing influenza in healthy adults (Review), *Cochrane Database of Systematic Reviews*: Reviews 2010 Issue 7 John Wiley & Sons, Ltd Chichester, UK DOI: 10.1002/14651858.CD001269.pub4

[16] Jefferson. T. et al. Vaccines for preventing influenza in the elderly (Review), *Cochrane Database of Systematic Reviews*: Reviews 2010 Issue 2 John Wiley & Sons, Ltd Chichester, UK DOI: 10.1002/14651858.CD005187.pub3

[17] Geoffery, A. W. & Perter, G. S., Vaccine Epidemiology: Efficacy, Effectiveness, and the Translational Research Roadmap, The Journal of Infectious Diseases 201, 1607-1610 (2010)

[18] Ministry of Health, Labour and Welfare Japan. Current standard vaccination schedule (estimation, as of December 16, 2009) (Japanese).
⟨http://www.mhlw.go.jp/kinkyu/kenkou/influenza/dl/infu091217-01.pdf⟩ (2009).

[19] William W. Thompson et al. Mortality Associated With Influenza and Respiratory Syncytial Virus in the United States. *JAMA* **289**(2), 179–186 (2003).

[20] Dawood, F.S. et al. Emergence of a novel swine-origin influenza A (H1N1) virus in humans, N. Engl. J. Med. 360 (25) (2009) 2605–2615.

[21] Tokyo metropolitan government. Tokyo Statistical Yearbook, 2-2 Population by 5 year age group and sex (1920–2005). ⟨http://www.toukei.metro.tokyo.jp/tnenkan/2008/tn08qyti0510b.htm⟩ (2008).
